# Anogenital Ulcers: An Unusual Manifestation of Invasive Aspergillosis

**DOI:** 10.1155/2018/7474135

**Published:** 2018-12-18

**Authors:** Pablo Vargas, Fernando Valenzuela, Viera Kaplan, Jacob Yumha, Montserrat Arceu, Claudia Morales

**Affiliations:** ^1^Department of Dermatology, Faculty of Medicine, University of Chile, Santiago, Chile; ^2^Faculty of Medicine, University of Chile, Santiago, Chile; ^3^Pathology Service, University of Chile Clinical Hospital, Santiago, Chile

## Abstract

*Aspergillus *spp. is one of the most ubiquitous fungi but generally does not cause disease in immunocompetent patients. It is the second most frequent agent of opportunistic fungal infections, after* Candida albicans*, with a rise in its incidence on recent years. Invasive fungal diseases represent an important cause of morbidity and mortality. Its origin can be primary, in relation to a cutaneous injury, or secondary, by extension from contiguous tissues, or by hematogenous spread, usually in the context of pulmonary aspergillosis. In this report, we describe the case of an elderly woman with invasive aspergillosis that manifested with anogenital and skin ulcers, with unfavorable outcome, despite intensive therapy and intravenous antifungals.

## 1. Introduction

Invasive fungal diseases represent an important cause of morbidity and mortality, mainly in immunocompromised patients.* Aspergillus *spp., after* Candida albicans*, is the second most frequent agent of opportunistic fungal infections, with an increase in its incidence on recent years [1, 2]. It is one of the most ubiquitous fungi but generally does not cause disease in immunocompetent patients [3]. Its most frequent portal of entry is the airway; therefore, pulmonary aspergillosis has been well characterized in the literature; however, the cutaneous form has not been that well characterized, probably because of its lower prevalence. Its origin can be primary, in relation to a cutaneous injury, or secondary, by extension from contiguous tissues, or by hematogenous spread, usually in the context of pulmonary aspergillosis [4]. We present the case of an elderly woman with invasive aspergillosis that manifested with anogenital and skin ulcers.

## 2. Case Report

**Figure 1:**
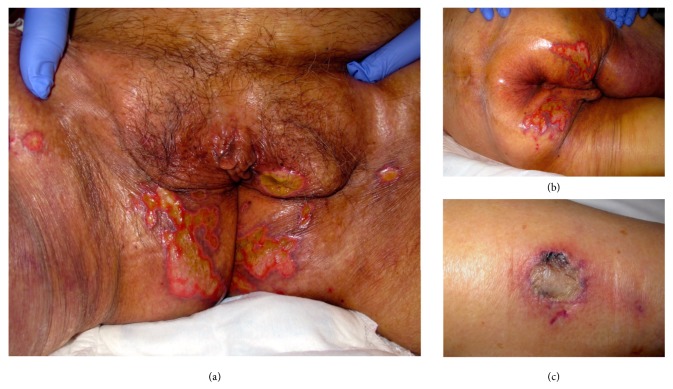
(a) Multiple painful genital and inguinal ulcers. (b) Ulcers extension to perianal region. (c), Ulcer in the right leg.

**Figure 2:**
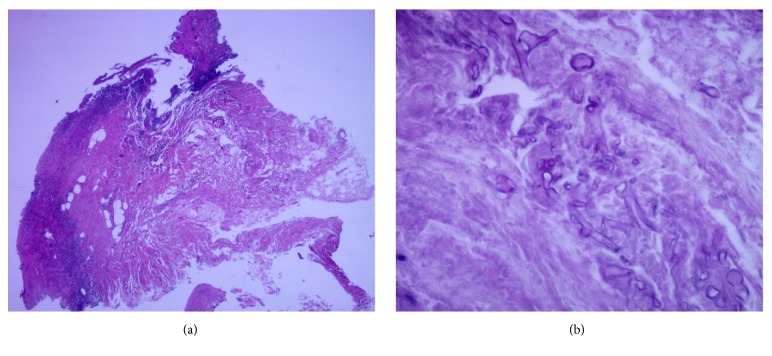
(a) 20x H/E. Skin and subcutaneous adipose tissue with ulcer and tissue necrosis. (b) 400x PAS. Septate and fragmented hyphaes of medium thickness in dermis with invasion of blood vessels.

An 82-year-old female patient is with a history of chronic arterial hypertension, ischemic stroke without sequelae, and hypothyroidism. She is hospitalized in our institution with a diagnosis of nephrotic syndrome, for study and management. Prednisone 1 mg/kg/day is started at admission. There was a torpid progression with multiple intercurrent infections, right renal infarction, and a progressive deterioration of kidney function, requiring the initiation of hemodialysis. In this context, after 3 weeks of hospitalization, she manifested multiple painful genital and inguinal ulcers, the largest one on the skin of the left labia majora, 1.5 cm in diameter, with a well-defined erythematous border and base with scarce fibrin. There was a rapid progression of the ulcers, with an increase in their size, number, and the extension to the perianal region, thighs, and right leg (Figure 1). Dermatology department was consulted, and polymerase chain reaction (PCR) for* herpes simplex viruses 1 and 2*,* Varicella zoster virus*,* Epstein barr virus*, and* Cytomegalovirus*, in addition to* HIV* serology and* VDRL*, were performed, with negative results. Biopsies of the vulvar and right leg lesions were taken and, on the PAS staining of the latter, septate hyphae were found, some with ramifications at acute angles and with invasion of blood vessels (Figure 2). Cultures of the lesions were negative. The patient presented with respiratory distress, and chest computed tomography showed a cavitated lesion in the upper segment of the left lower lobe, suggestive of aspergilloma. Galactomannan blood test came back positive, thus confirming the diagnosis of invasive aspergillosis. Intravenous antifungal therapy with voriconazole and caspofungin was initiated; however, the patient deteriorated rapidly, with multiorgan failure, and died despite intensive care and twenty days of antifungal treatment.

## 3. Discussion

Invasive aspergillosis affects the skin in 1 to 10% of cases, according to some retrospective series, and it is the second most frequent site after the lung [5, 6]. It usually manifests with papules and nodules on the trunk and limbs. It can evolve with necrosis and ulceration [6], the latter being more frequent in cases of primary aspergillosis. In the main retrospective series, no cases of aspergillosis with anogenital involvement have been reported [5–7], and only one case is described, which corresponds to a primary cutaneous form [8]. The development of this fungal infection usually occurs in immunocompromised patients, mainly in hemato-oncological patients. Other independent risk factors are recognized, among which advanced age stands out and also leads to higher mortality [9]. Our patient was elderly and, in addition, immunocompromised by the use systemic corticosteroids due to the nephrotic syndrome. Considering the low sensitivity of culture techniques, it is necessary to integrate the clinic with diagnostic tools. For some authors, histopathological study represents the gold standard for diagnosis, even though microscopic differentiation from other filamentous fungi can be difficult. There are some diagnostic clues, such as the branching of hyphae at acute angles, vascular invasion, or thrombosis [8, 9]. There is high quality evidence to recommend serum galactomannan in the diagnosis and follow up of invasive aspergillosis in adult and pediatric patients. Also, PCR techniques are controversial and their use should be evaluated according to the conditions of each patient [8]. Voriconazole is the first-line treatment for invasive aspergillosis, and it is suggested that it should be used along with caspofungin in the case of pulmonary compromise [9, 10].

We present this case given the unusual presentation of invasive aspergillosis with anogenital ulcers, and it should be considered in the differential diagnosis of these kinds of lesions in the immunocompromised.
